# Expanding the clinicopathological-genetic spectrum of glycogen storage disease type IXd by a Chinese neuromuscular center

**DOI:** 10.3389/fneur.2022.945280

**Published:** 2022-08-11

**Authors:** Kun Huang, Hui-Qian Duan, Qiu-Xiang Li, Yue-Bei Luo, Fang-Fang Bi, Huan Yang

**Affiliations:** ^1^Department of Neurology, Xiangya Hospital, Central South University, Changsha, Hunan Province, China; ^2^National Clinical Research Center for Geriatric Disorders, Xiangya Hospital, Central South University, Changsha, Hunan Province, China

**Keywords:** PHKA1, glycogen storage disease type IXd, neuromuscular disorder, glycogen storage disease, myopathy

## Abstract

**Background:**

Glycogen storage disease (GSDs) is characterized by abnormally inherited glycogen metabolism. GSD IXd, which is caused by mutations in the *PHKA1* gene, is an X-linked rare disease with mild myopathic symptoms. To date, only 13 patients with GSD IXd have been reported. In this study, we aimed to expand the clinicopathological-genetic spectrum of GSD IXd at a neuromuscular center in China.

**Methods:**

Data on patients diagnosed with GSD IXd at our neuromuscular center were collected retrospectively. Clinical features, electrophysiology, muscle pathology, and genetic information were analyzed.

**Results:**

Between 2015 and 2021, three patients were diagnosed with GSD IXd based on clinical manifestations, pathological findings, and genetic testing. One patient presented with mitochondrial myopathy. All patients exhibited muscle weakness and elevated levels of creatine kinase. Electromyography-detected myopathic changes were found in two patients, whereas one patient refused to undergo this examination. Pathological examinations in all patients revealed subsarcolemmal accumulation of glycogen under PAS staining. All patients had mutations in the *PHKA1* gene and the patient with mitochondrial myopathy also had a mutation in the *MT-TL1* gene.

**Conclusion:**

Our study expands the clinicogenotype and phenotype of GSD IXd in a Chinese population. Our study also expands the known mutation spectrum for GSD IXd, contributing to a better characterization and understanding of this ultrarare neuromuscular disorder.

## Introduction

Glycogen storage diseases (GSDs) are characterized by abnormally inherited glycogen metabolism in the liver, muscles, and brain. GSDs are divided into different types, from 0 to X. GSD IX is a rare disease of variable clinical severity, primarily affecting the liver or skeletal muscles. GSD IX is caused by deficient phosphorylase kinase (PhK) (1). PhK is a hexadecameric complex, (αβγ*δ*)_4_, whose subunits have several isoforms, each encoded by a different gene, generating distinct tissue-specific mRNA splice variants (2). The enzyme subunits of PhK are encoded by various genes: α (*PHKA1, PHKA2*), β (*PHKB*), γ (*PHKG1, PHKG2*), and δ (*CALM1, CALM2, CALM3*) (Table 1). GSD IX is classified into a, b, c, d four subtypes. GSD IXa, GSD IXb and GSD IXc are caused by mutations in *PHKA2* (3), *PHKB* (4), and *PHKG2* (5), respectively. GSD IXd is caused by mutations in *PHKA1* or *PHKG1* (6, 7).

**Table 1:** Phosphorylase kinase (PhK) subunit genes.

**Gene**	**PhK subunit**	**Location**	**Inheritance**	**Tissue involved**
*PHKA1*	α	Xq13.1	XL^*^	Muscle
*PHKA2*	α	Xp22.13	XL	Liver
*PHKB*	β	16q12.1	AR^*^	Liver
*PHKG2*	γ	16q11.2	AR	Liver
*PHKG1*	γ	7p11.2	AR	Muscle
*CALM1*	δ	14q32.11	AD^*^/AR	Ubiquitously expressed
*CALM2*	δ	2p21	AD/AR	Ubiquitously expressed
*CALM3*	δ	19q13.32	AD/AR	Ubiquitously expressed

* XL, X-linked; AR, autosomal recessive; AD, autosomal dominant.

PhK deficiency underlies a wide spectrum of glycogenoses. Liver PhK deficiency alone accounts for approximately 25% of all GSD cases (8). Muscle PhK deficiency appears to be ultrarare but could be underdiagnosed due to the milder and more variable muscle symptoms. These entities are differentiated depending on their inheritance patterns and affected tissues. PhK plays a key role in glycogen metabolism because it catalyzes the phosphorylation of a serine residue on glycogen phosphorylase (9). PhKs consist of regulatory subunits α and β, catalytic subunit γ, and a calcium sensitivity subunit with calmodulin δ. The α-subunit consists of two isoforms: the muscle-specific variant (αM), which is the product of the *PHKA1* gene on chromosome Xq13.1, and the liver isoform (αL), encoded by *PHKA2* on chromosome Xp22.13 (10, 11).

Mutations in *PHKA1* have been reported to cause GSD IXd, an X-linked ultra-rare disease with mild myopathic symptoms, including exercise intolerance, cramps, myalgia, weakness, and myoglobulinuria (6). To the best of our knowledge, only 13 patients with GSD IXd have been reported to have mutations in the *PHKA1* gene (12, 13). In this article, we describe three patients from unrelated families with GSD IXd, with manifestations of exercise intolerance. *PHKA1* mutations were confirmed in these three patients. Muscle biopsies were conducted and muscle pathology was analyzed. Specifically, one patient with GSD IXd had mitochondrial myopathy with mtDNA mutation (m.3243A>G). Our research expands the spectrum of GSD IXd due to *PHKA1* mutations in a Chinese neuromuscular center.

## Materials and methods

### Ethics approval

Ethical approval was granted by the Ethics Committee of Xiangya Hospital, Central South University. Informed consent for participation in our study was obtained from all patients, as previously reported by our center (14).

### Patients and clinical evaluation

Between 2015 and 2021, three patients were diagnosed with GSD IXd based on clinical manifestations, pathological findings, and genetic testing at the neuromuscular center of Xiangya Hospital, Central South University. Clinical assessments of the patients consisted of physical examinations and laboratory investigations, such as serum creatine kinase (CK), electromyography, muscle biopsy, and genetic testing, as previously used in our center (15, 16).

### Pathological examination of biopsy specimens

Open muscle biopsy specimens were obtained from the left gastrocnemius or biceps brachii. Muscle samples were immediately frozen in isopentane, cooled with liquid nitrogen, and stored at −80°C. Immunohistochemical staining was performed as described previously with minor modifications (17–20). Briefly, histological and immunohistochemical analyses were performed on 8 μm-thick frozen sections prepared using a cryostat. For muscle sections, routine histological examinations included staining with hematoxylin and eosin (HE), modified Gömöri trichrome, periodic acid-Schiff (PAS), oil red O, acid phosphatase, NADH-tetrazolium reductase, ATPase (pH 4.3, 4.6, and 11.0), and succinic dehydrogenase. Immunohistochemistry was performed using antibodies against dystrophin and dysferlin.

### Genetic testing

Genomic DNA was extracted using DNeasy Blood and Tissue Kits (Qiagen, Venlo, Netherlands) according to the manufacturer's instructions, and the samples were sent to Guangzhou Jiajian Medical Testing Co., Ltd. (Guangzhou, China) or Beijing MyGenostics Co. Ltd. (Beijing, China). Next-generation sequencing analysis, which targeted the exons and exon–intron junctions of genes known to be associated with hereditary neuropathies and myopathies, was performed. The samples were pooled and sequenced on a HiSeq X Ten (Illumina, San Diego, CA, USA) using 2 × 150 paired-end sequencing. Sequences were aligned to the human genome reference (hg19) sequence using the Burrows–Wheeler Alignment tool (BWA 0.7.12) with default parameters. Detected sequence variants, if present in the dbSNP, HapMap, 1000 Genome, ESP6500, ExAC, or in-house Chinese Exome Database (1500 Chinese Han individuals), were removed. Sanger sequencing was performed to confirm *PHKA1* mutations. The possible impact of nonsense mutations was predicted using MutationTaster (https://www.mutationtaster.org) and splicing mutations were predicted using NNSplice (http://www.fruitfly.org/seq_tools/splice.html).

## Results

### Clinical features

#### Patient 1

Patient 1 was a 41-year-old man who was born to non-consanguineous parents. The patient had no family history of muscle disease. One year earlier, he had experienced chest distress and presented to the cardiology department. His CK level was 8,028 U/L (normal range 50–310 U/L), and troponin levels and electrocardiogram result were normal. The patient refused any medications. One month prior, the patient had experienced gradual muscle weakness and myalgia of all four limbs. The patient had lost 10 kg of weight during this year. Physical examination revealed a positive Gower's sign. Tendon reflexes were decreased in the upper limbs and had disappeared in the lower limbs. The Hoffmann, Babinski, and Chaddock signs were bilaterally negative. Muscle strength testing revealed weakness of the iliopsoas (3/3 on the MRC scale [left/right]), proximal and distal upper limbs (4/4) and proximal and distal lower limbs (4/4). Retesting of serum CK levels showed a high increase (17060 U/L). Electromyography (EMG) revealed myopathic changes.

#### Patient 2

Patient 2 was a 31-year-old man born to non-consanguineous parents. The patient denied any family history of muscle disease. Six months prior, he had muscle weakness of the lower limbs and gradually lost the ability to climb stairs. Tendon reflexes were normal. The Hoffmann, Babinski, and Chaddock's signs were negative. Muscle strength testing revealed weakness of the cervical muscles (3/3), proximal upper limbs (4/4), and proximal lower limbs (4/4). The serum CK level was highly elevated (9,200 U/L). EMG revealed myopathic changes.

#### Patient 3

Patient 3 was a 13-year-old boy born to non-consanguineous parents. The patient had no family history of muscle disease. The patient was undersized after birth and his muscle strength was less than that of his classmates during childhood. Two months prior, he had swelling of the lower limbs associated with muscle weakness of the lower limbs. The Gowers' sign was positive. Tendon reflexes were normal. The Hoffmann, Babinski, and Chaddock signs were bilaterally negative. Muscle strength testing revealed weakness of the cervical muscles (4/4), iliopsoas (3/3), proximal upper limbs (4/4), and proximal and distal lower limbs (4/4). The serum CK level was moderately increased (621 U/L). Plasma lactate was elevated at rest (2.06 mmol/L, normal range 1.42–1.90 mmol/L). The patient refused to undergo EMG.

### Muscle pathology

**Figure 1:**
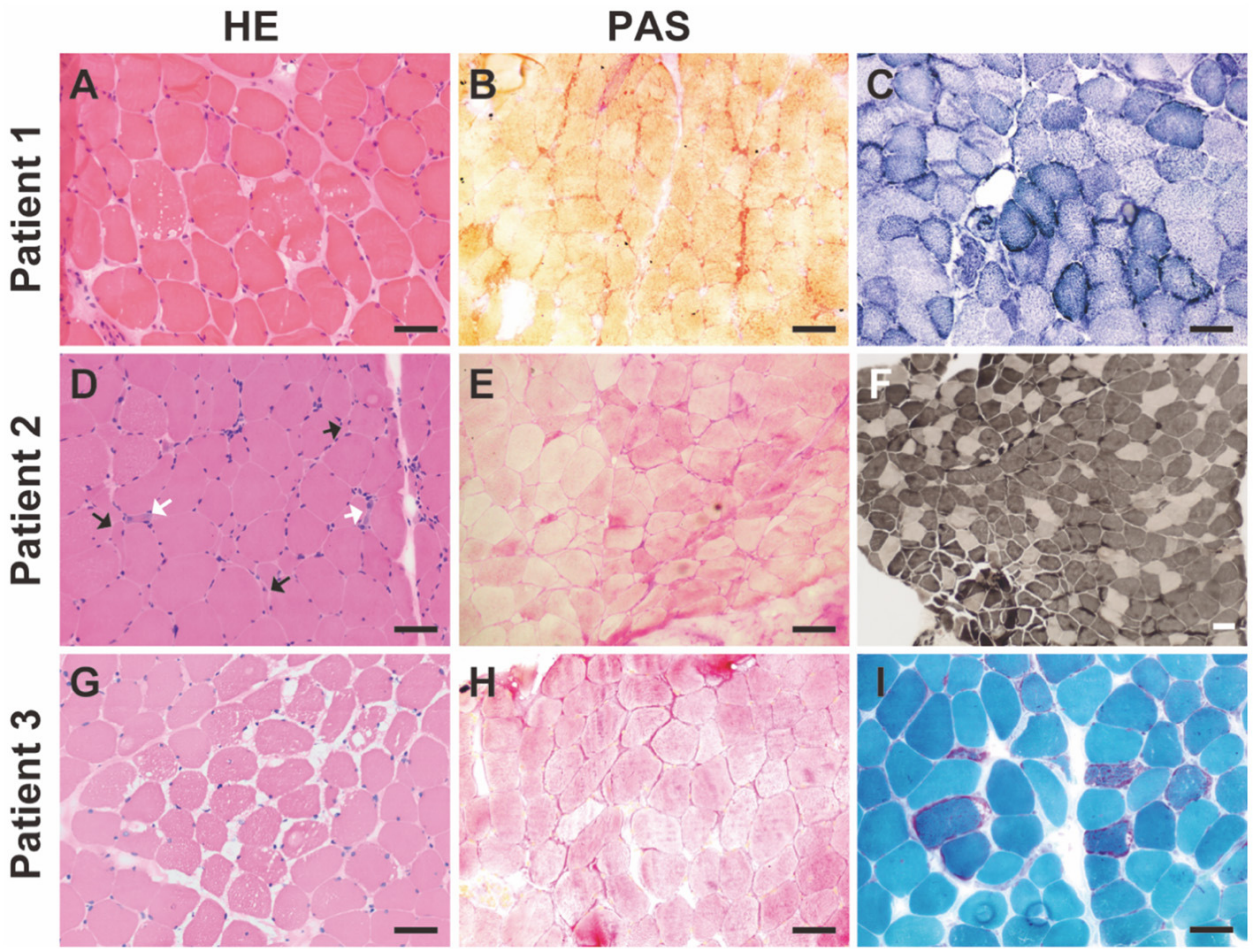
Myopathological changes in the patients with GSD IXd. **(A)** Hematoxylin-Eosin (HE) staining shows increased fiber size variation, slight hyperplasia of connective tissue, and vacuoles. **(B)** Periodic acid-Schiff (PAS) staining shows subsarcolemmal accumulations of glycogen. **(C)** NADH staining shows slight myofibrillar network disarray and increased subsarcolemmal mitochondria in a subset of myofibers. **(D)** HE staining shows increased fiber size variation, scattered angular fibers (black arrows), and regenerative fibers (white arrows). **(E)** PAS staining shows subsarcolemmal accumulations of glycogen. **(F)** ATPase staining (pH 4.2) shows type I myofiber predominance. **(G)** HE staining shows increased fiber size variation, slight hyperplasia of connective tissue and vacuoles. **(H)** PAS staining shows subsarcolemmal accumulations of glycogen. **(I)** Modified Gömöri staining shows red-ragged fibers. Scale bar = 50 μm.

Muscle biopsies from all three patients were examined. Pathology revealed GSD changes in all three patients with different features (Figure 1). In addition, changes in mitochondrial myopathy, such as ragged-red fibers, were observed in Patient 3 (Figure 1). Common myopathological changes in these three patients included increased fiber size variation, scattered regenerative myofibers, and subsarcolemmal accumulation of glycogen (PAS staining). Other myopathological changes, such as type I myofiber predominance, were observed in Patient 2, and ragged-red fibers, ragged-blue fibers, and myofibrillar network disarray were detected in Patient 3. Further details of muscle biopsies are presented in Table 2. Immunohistochemical analysis of dystrophin-R, dystrophin-C, dystrophin-N, sarcoglycan-α, sarcoglycan-β, sarcoglycan-γ, sarcoglycan-δ, and dysferlin showed no abnormalities.

**Table 2:** Summary of patients' examinations.

**Patient**	**CK (U/L)**	**EMG**	**Genotype**	**Muscle biopsy**
1	17060	Myopathic changes	*PHKA1*: c.1533T>A (p.Y511*)	IFSV, scattered necrotic fibers, regeneration, slight hyperplasia of connective tissue, vacuoles (HE staining), subsarcolemmal accumulations of glycogen (PAS staining)
2	9200	Myopathic changes	*PHKA1*: c.3297 + 5G>A	IFSV, scattered angular fibers, regenerative fibers, type I myofiber predominance, subsarcolemmal accumulations of glycogen (PAS staining)
3	621	NA	*MT-TL1*: m.3243A>G *PHKA1*: c.3670_3924del255(skips exon 29 and 30)	IFSV, scattered atrophic fibers, slight hyperplasia of connective tissue, vacuoles (HE staining), myofibrillar network disarray, RRF (modified Gömöri staining), RBF (SDH staining), subsarcolemmal accumulations of glycogen (PAS staining)

CK, the latest creatine kinase before muscle biopsy; normal range: 50–310 U/L; EMG, electromyography; IFSV, increased fiber size variation; NA, not available, RRF, ragged-red fiber; RBF, ragged-blue fiber.

### Genetic testing and mutations

Mutation analysis of genes known to be associated with hereditary neuropathies and myopathies was performed. All patients were confirmed to have mutations in *PHKA1* (NM_002637 for all patients) (Table 2). Patient 1 had a nonsense mutation of c.1533T>A (p.Y511^*^), causing the deletion of functional domains, including the two calmodulin-binding domains. MutationTaster predicted the c.1533T>A (p. Y511^*^) mutation to be disease-causing (score:6.0). Patient 2 had a splicing mutation c.3297 + 5G>A. Patient 3 had a deletion mutation of c.3670_3924del in *PHKA1*, which caused the deletion of exons 29 and 30. In addition, whole-mitochondrial genome analysis of patient 3 identified a mutation in m.3243A>G in the *MT-TL1* gene with a heteroplasmy of 87.1%. The mother of patient 3 exhibited a mutation in m.3243A>G, with a heteroplasmy of 1.2%. All the mutations of *PHKA1* in our study were classified as “likely pathogenic” according to the American College of Medical Genetics and Genomics standard guidelines (21).

## Discussion

The *PHKA1* gene encodes the muscle isoform of the α subunit (αM) of phosphorylase b kinase (22). GSD IXd, caused by mutations in the *PHKA1* gene, is an ultra-rare type of mild myopathy with exercise intolerance. To date, only 13 patients with GSD IXd have been reported, most of whom presented with exercise intolerance and mild-to-moderate myopathy (Table 3). Since the number of reported patients is too small, it is difficult to perform further phenotypic analyses. There is no consistent genotype-phenotype correlation for pathogenic variants of *PHKA1*. The age of onset ranged across all ages. The symptoms are varied, from muscle weakness with only proximal limb or distal limb involvement to only cognitive impairment or an isolated increase in CK level without overt symptoms of myopathy (23). In the current study, we identified three GSD IXd patients with *PHKA1* mutations from three unrelated Chinese families and analyzed their clinical features, muscle pathology, and genetic variants in detail. Specifically, one patient with GSD IXd had mitochondrial myopathy, which has never been reported in a patient with GSD IXd.

**Table 3:** Patients with GSD IXd harboring mutations in the *PHKA1* gene.

**Case**	**Sex/ Age(year)**	**Age at onset (year)**	**Chief complaint**	**CK**	**EMG**	**Mutation**	**Muscle biopsy**	**Reference**
1	M/64	46	gait disturbance	2N^*^	Myopathic changes	c.3334G>T (p.E1112^*^)	subsarcolemmal accumulations of glycogen	(24)
2	M/18	6	exercise intolerance	6N	Myopathic changes	c.896A>T (p.D299V)	subsarcolemmal accumulations of glycogen	(25)
3	M/28	15	exercise intolerance	3N	Mild myopathic changes	c.3498 + 1G>C (p.M1100_Q1166del)	subsarcolemmal accumulations of glycogen	(26)
4	M/56	0.5	myalgia	10N	Normal	c.695delC (p.A232fs)	subsarcolemmal accumulations of glycogen	(27)
5	M/50	Childhood	exercise intolerance	2N	NA	c.831>A (p.G223R)	NA	(28)
6	M/17	17	hyperCKemia	5N	Mild myopathic changes	c.1394delT (p.T464fs)	subsarcolemmal accumulations of glycogen	(23)
7	M/39	32	myalgia	1.7N	Myopathic changes	c.1293delT (p.F430fs)	subsarcolemmal accumulations of glycogen	(29)
8	M/69	64	hyperCKemia	5N	Normal	c.695delC (p.A232fs)	subsarcolemmal accumulations of glycogen	(29)
9	M/33	NA	myalgia, fatigue	Elevated	NA	c.586G>A (p.E196K)	subsarcolemmal accumulations of glycogen	(30)
10	M/25	15	muscle weakness	Elevated	Normal	c.3246T>A (p.C1082^*^)	subsarcolemmal accumulations of glycogen	(7)
11	M/55	40	exercise intolerance, myalgia	20N	Normal	c.2594delA (p.K865Rfs^*^15)	subsarcolemmal accumulations of glycogen	(12)
12	F/73	72	camptocormia	2N	Normal	c.2594delA (p.K865Rfs^*^15)	NA	(12)
13	M/16	15	myalgia	Elevated	NA	c.3579_3580insT (p.S1194^*^)	subsarcolemmal accumulations of glycogen	(13)

* 2N: two-fold of normal range.

Although the symptoms of GSD IXd vary, elevated CK levels may be one of the first and most consistent findings in patients with GSD IXd. All previously reported patients with GSD IXd, as well as patients in our study, displayed slightly to highly elevated CK levels. One reported patient with GSD IXd, who had only cognitive impairment without overt myopathy, presented with five-fold increased CK levels (23). In our study, Patient 1 first experienced chest distress and had a highly increased CK level (8,028 U/L) without any overt myopathy symptoms, such as muscle weakness or myalgia. As the disease progressed, the patient gradually developed muscle weakness and myalgia. The medical history of patient 1 indicated that elevated CK levels may be the primary symptom of GSD IXd prior to other symptoms, such as muscle weakness.

As most activities in daily life do not require intense exercise, myophosphorylase is not fully activated in patients with GSD IXd (29). The muscle PhK deficiency and insufficient glycogenolysis in GSD IXd patients do not translate into symptoms at low intensities of daily life, but it likely explains the mild glycogen accumulation observed in patients with GSD IXd. Elevated CK levels may be caused by minimal membrane damage as a result of intermittent energy shortage in myofibers (31).

Currently, little is known about the precise pathophysiological mechanism of *PHKA1* mutations in GSD IXd. *In vivo*, the *phka1*-deficient I-strain mouse exhibited almost no expression of *Phka1*, resulting in loss of the ability to associate with the other Phk subunits to form the holoenzyme complex (32). Another *phka1*-deficient I/LnJ mouse exhibited half-decreased running time and distance compared to wild-type mice (33). However, the transcriptional expression of key enzymes necessary for glucose transport and glycolytic flux was not significantly different between *phka1*-deficient I/LnJ mice and wild-type mice, suggesting that non-glycolytic mechanisms work to maintain muscle function in *phka1*-deficient I/LnJ mice (33).

There have been no reports of the co-occurrence of mitochondrial myopathy and GSD. One of our patients (patient 3) had an unusual case of GSD IXd accompanied by mitochondrial myopathy. Compared to the other two patients (patients 1 and 2), patient 3 showed an earlier onset in childhood, suggesting that the symptoms of GSD IXd accompanied by mitochondrial myopathy were more severe than those of patients with GSD IXd only. Although rare, co-occurrence of GSD IXd with other neuromuscular disorders may cause phenotypic alterations.

Currently, little is known about the phenotypic features of GSD IXd patients with *PHKA1* mutations. This condition may progress gradually and become evident later in life. Our study expands the clinicogenotype and phenotype of GSD IXd in the Chinese population. Our study also expands the known mutation spectrum of GSD IXd, contributing to a better characterization and understanding of this ultrarare muscular disorder.

## Data availability statement

The original contributions presented in the study are included in the article/supplementary files, further inquiries can be directed to the corresponding author/s.

## Ethics statement

Ethics approval was granted by the Ethics Committee of Xiangya Hospital, Central South University. The patients/participants provided their written informed consent to participate in this study.

## Author contributions

KH and HY conceived the project. KH, H-QD, and Q-XL acquired and analyzed the data with the help of Y-BL, F-FB, and HY. KH wrote the manuscript. HY critically revised the manuscript for valuable intellectual content. All authors approved the final manuscript.

## Funding

This work was supported by the Science and Technology Innovation Program of Hunan Province, China (Grant No. 2021RC2023, KH), and the China Postdoctoral Science Foundation (Grant No. 2021M703638, KH).

## Conflict of interest

The authors declare that the research was conducted in the absence of any commercial or financial relationships that could be construed as a potential conflict of interest.

## Publisher's note

All claims expressed in this article are solely those of the authors and do not necessarily represent those of their affiliated organizations, or those of the publisher, the editors and the reviewers. Any product that may be evaluated in this article, or claim that may be made by its manufacturer, is not guaranteed or endorsed by the publisher.
